# Honeycomb Cell Structures Formed in Drop-Casting CNT Films for Highly Efficient Solar Absorber Applications †

**DOI:** 10.3390/nano14201633

**Published:** 2024-10-11

**Authors:** Saiful Islam, Hiroshi Furuta

**Affiliations:** 1 School of Systems Engineering, Kochi University of Technology, Kochi 782-8502, Japan; md.saiful38@gmail.com; 2 Center for Nanotechnology, Research Institute, Kochi University of Technology, Kochi 782-8502, Japan

**Keywords:** solar absorber, spectral selectivity, honeycomb structure, drop-casting technique, CNTs, solar radiation intensity

## Abstract

This study investigates the process of using multi-walled carbon nanotube (MWCNT) coatings to enhance lamp heating temperatures for solar thermal absorption applications. The primary focus is studying the effects of the self-organized honeycomb structures of CNTs formed on silicon substrates on different cell area ratios (CARs). The drop-casting process was used to develop honeycomb-structured MWCNT-coated absorbers with varying CAR values ranging from ~60% to 17%. The optical properties were investigated within the visible (400–800 nm) and near-infrared (934–1651 nm) wavelength ranges. Although fully coated MWCNT absorbers showed the lowest reflectance, honeycomb structures with a ~17% CAR achieved high-temperature absorption. These structures maintained 8.4% reflectance at 550 nm, but their infrared reflection dramatically increased to 80.5% at 1321 nm. The solar thermal performance was assessed throughout a range of irradiance intensities, from 0.04 W/cm^2^ to 0.39 W/cm^2^. The honeycomb structure with a ~17% CAR value consistently performed better than the other structures by reaching the highest absorption temperatures (ranging from 52.5 °C to 285.5 °C) across all measured intensities. A direct correlation was observed between the reflection ratio (visible: 550 nm/infrared: 1321 nm) and the temperature absorption efficiency, where lower reflection ratios were associated with higher temperature absorption. This study highlights the significant potential for the large-scale production of cost-effective solar thermal absorbers through the application of optimized honeycomb-structured absorbers coated with MWCNTs. These contributions enhance solar energy efficiency for applications in water heating and purification, thereby promoting sustainable development.

## 1. Introduction

Global development and human progress are significantly impeded by the scarcity of potable water, electricity, and heated water for domestic and industrial applications [1,2,3]. Electricity generation, water heating, and seawater desalination are among the numerous applications of solar energy, which is distinguished by its abundance, accessibility, and reliability. Nevertheless, the thermal efficacy of solar energy systems remains suboptimal despite these advantages. A promising approach to enhancing the efficacy of solar thermal devices is enhancing the properties of solar absorption materials [4]. Hydrophilicity, porosity, self-floating properties, and high solar thermal conversion efficiency are all essential characteristics of ideal solar absorption materials [5]. These materials are essential for solar applications because they capture solar radiation and convert it to thermal energy, which is transferred to a working fluid. Carbon nanotubes (CNTs) have become highly effective solar absorbers because of their broad absorption spectrum [6]. CNTs, which were first identified by Sumio Iijima in 1991 [7], have attracted attention for their exceptional optical [8], electrical [9], and mechanical [10] properties. Their exceptional light-absorbing capabilities render them one of the darkest materials [11]. The vital functions of surface micro–nano structure design and preparation procedures in determining absorption performance in practical applications are underscored by the inherent absorption properties of MWCNT film [12,13,14,15]. The broadband absorption characteristics of the MWCNT absorber, which include the infrared (IR) region, contribute to increased radiation loss, even though MWCNTs exhibit high thermal conductivity and exceptional optical absorption properties [16,17].

Previous research has introduced a variety of structures, such as multilayered formations and composite absorbers, to resolve wavelength selectivity and radiation issues [6,18]. This comprehension underscores the significance of modifying the structural characteristics of MWCNT film-based absorbers (such as those with a 3D wavy structure [19], a peak-like structure [20], a forest-like structure [21], or a multilayer structure [22]) to optimize their efficacy in solar absorbers. Udorn et al. [23] reported that CNT honeycomb structures, which were fabricated by short ethanol treatment durations on CNT forests (synthesized via thermal CVD, yielding ~8.0 × 10^9^ CNTs/cm^2^ with ~10 µm height) and had cell areas below 30 µm^2^, wall heights around 5.5 µm, and thin buckypaper films (0.2 µm), exhibited a high total reflectance of up to 10–12% in the UV region and 6–8% in the visible region. Hong et al. [24] developed the 3D origami solar steam generator by spray coating a graphene oxide/carbon nanotube composite onto a preheated porous cellulose membrane structured with a Miura-ori tessellation. The generator achieved a surface area density of 4.65 and an evaporation rate of 1.59 kg/(m^2^ h) under one period of sun illumination, resulting in a solar energy efficiency of nearly 100%. Kiani et al. [25] prepared a hierarchical Cu-CNT nanowire structure fabricated through copper thermal oxidation, hydrogen reduction, and self-catalytic CVD of CNTs using acetylene. The resultant structure exhibited ultra-broadband near-perfect light absorption, with an average total reflectance of 0.75% and a specular reflectance of 0.1% over the 400–1000 nm wavelength range. Ghai et al. [26] synthesized flower-like carbon nanotubes (FCNTs) using a two-step thermal CVD process that involved a dual etching technique. These FCNTs demonstrated exceptional blackbody properties, with a light absorption rate of over 99.97% and an emissivity of 0.98 in the UV–vis–NIR range. Pander et al. [27] demonstrated that a CNT forest made with fishnet metamaterials, which were self-assembling and used a polystyrene nanosphere monolayer as a shadow mask during the deposition of the catalyst, showed a blueshift in its broadband reflectance peak from 550 nm to 460 nm, as the size of the holes increased from 370 nm to 665 nm, which was attributed to changes in inductance and capacitance. Huang et al. [28] prepared a multilayered structured absorber utilizing CMP–CNT (average tube diameter of CNT—15 nm)/CNP–TiN coatings and achieved an average light absorptance of 96.4% in the 400–1400 nm wavelength range. The fabrication process involved the use of a high-voltage electrostatic spraying approach.

The thermal absorption of MWCNT-based absorbers is significantly improved under different solar intensities. It is essential to mention intensity issues because variations in solar irradiance directly influence the performance and efficiency of solar absorbers [29]. For instance, He et al. [30] prepared a polydopamine-encapsulated carbon nanotube/polyurethane (PDA@CNT/PU) nanofiber membrane solar steam generator, which achieved high evaporation rates and solar-to-vapor conversion efficiencies of up to 1.44 kg m^−2^ h^−1^ and 90.1% under one period of sun illumination, and exhibited excellent anti-oil-fouling ability and stability even with oil-contaminated water. Jin et al. [31] reported that the carbon nanotubes and reduced graphene oxide deposited onto a bacterial cellulose (CNT-RGO@BC) composite absorber exhibited efficient solar interface evaporation, as evidenced by the absorber’s high photothermal conversion efficiency of 90.2% and evaporation rate of 1.85 kg m^−2^ h^−1^ under one period of solar irradiation. Li et al. [32] demonstrated that their porous Ni/CNTs composite membrane, which was employed as a highly efficient solar absorber, achieved an impressive solar energy utilization rate of 94.3% and an evaporation rate of 2.13 kg m^−2^ h^−1^ under one period of sun irradiation, with a radiation loss of 1.6%. Zhang et al. [33] introduced a spray-based method for the bulk production of GO/CNT-based solar evaporator membranes. They utilized a tunnel to continuously dry the membrane evaporator. They achieved inadequate efficiency as the sun’s intensity increased due to heat losses. Wang et al. [34] introduced a hanging mode solar evaporator, which utilized a polyaniline/carbon nanotube (PANI/CNT) composite absorber and obtained a photothermal efficiency of 91.74% and an evaporation rate of 2.81 kg·m^−2^·h^−1^ under one period of solar illumination.

Despite their promising features, the widespread adoption of MWCNT coatings in scalable applications is hindered by numerous inherent limitations. The fabrication methods for MWCNTs are relatively complex, frequently necessitating subsequent transfer processes and typically involving chemical vapor deposition (CVD) techniques. These procedures are mainly restricted to small-scale processes, substantially hindering the broader implementation and scale-up of MWCNT-based absorbers [35,36]. Additionally, MWCNT composite absorbers require high-cost and complex preparation procedures for large-scale fabrication. Furthermore, most experiments have been conducted in environments with 0.1 W/cm^2^ of sunlight (1 sun). However, illumination levels higher than this are required for practical applications due to the variation in solar irradiance from one region to another, which is influenced by the weather and climate. The actual applications of solar thermal devices in environments with low (<0.1 W/cm^2^) or high (>0.1 W/cm^2^) radiation have substantial effects [37,38].

In this paper, the relationship between self-organized honeycomb-structured MWCNT absorbers and the optical and thermal properties of MWCNT honeycombs is reported. This investigation introduces a simple method for fabricating randomly aligned MWCNT honeycombs. MWCNT honeycomb structures with different cell areas and fully coated areas are presented. Subsequently, the reflectance of different cell areas of honeycomb structures composed of MWCNT is studied. Furthermore, the thermal performance of MWCNT honeycomb structures under different lamp intensities is evaluated. These findings are considered in the context of potential applications in solar thermal devices.

## 2. Methods

### 2.1. Materials

Randomly aligned multi-walled carbon nanotubes (MWCNTs) (5 wt%) dispersed in ethanol solvent (product name: MW-I) were purchased from Meijo Nanocarbon Co., Ltd. (Tokyo, Japan). The MWCNTs had a diameter ranging from 10–40 nm [39]. Ethanol (99.5% purity) was purchased from Wako Pure Chemical Industries, Ltd. (Osaka, Japan) and used without further purification. Thermally oxidized Si (th-SiO) wafers (SEMITEC, Chiba, Japan) were used for the experiments. These wafers had a SiO_2_ thickness of 100 nm ± 10 nm, a wafer thickness of 525 µm ± 25 µm, and a resistivity of ≥100 Ω·cm. The surface roughness of the th-SiO substrate was measured at 0.8 nm.

### 2.2. Preparation of MWCNTs Absorber

**Figure 1:**
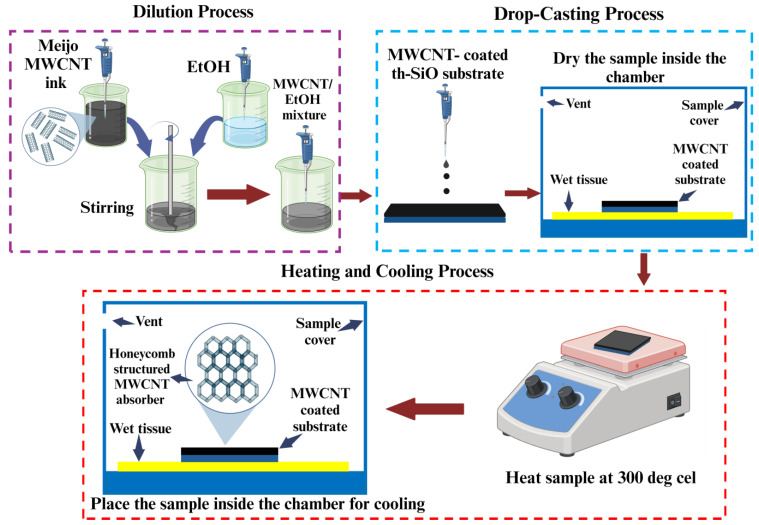
Detailed procedure for preparing MWCNT-coated absorbers.

A fixed volume of 25.0 µL of dispersed MWCNTs was used for the preparation of each sample. Ethanol (EtOH) volumes ranged from 25 µL to 1000 µL. The range of concentrations was set as 25 µL for EtOH volumes ranging from 25 µL to 100 µL and 100 µL for EtOH volumes ranging from 100 µL to 1000 µL. To ensure homogeneity, set volumes of MWCNT solution and EtOH were combined and stirred for 2 min for each sample. The thermally oxidized Si (th-SiO) substrates with a resistivity of ≥100 Ω·cm were manually cut into 1 cm × 1 cm × 0.0525 ± 0.0025 cm using a diamond cutter and coated with 4.0 ± 0.5 µL of the prepared MWCNT/EtOH mixture using a drop-casting technique. The samples were subsequently placed inside a chamber with a temperature of ~25 °C and ~50% humidity for ~4 h to allow the EtOH solvent to evaporate. The bottom area of the chamber was covered by wet tissue to maintain the humidity at ~50%. Following this, each sample was subjected to a thermal treatment at 300 °C for 1 h to eliminate residual solvents and organic components. The heated samples were then placed back into the chamber to reach a temperature of 25 °C. The comprehensive procedure for preparing MWCNT-based absorbers is illustrated in Figure 1.

### 2.3. Optical Measurements

A UV–vis spectrophotometer (HITACHI U-3900, HITACHI high-technologies, Tokyo, Japan) provided with an integrating sphere was employed to measure the reflectance in the UV–visible wavelength region within the range of 190–900 nm. An optical spectrometer (USB2000+, Ocean Optics, Orlando, FL, USA) was deployed to measure specular reflectance in the visible range from 400 nm to 800 nm, using a 45-degree incidence angle. An IR spectrometer (FLAME NIR, Ocean Optics, USA) was employed to measure reflectance in the near-infrared region from 934 nm to 1651 nm. The NIR spectrometer was equipped with a tungsten halogen light (HL-2000-HP, Ocean Optics, USA) and a fiber optic holder (RPH-SMA, Thorlabs, Newton, NJ, USA) to detect reflectance in the near-infrared (NIR) range. The absorption spectrum was derived from the reflectance values using the following equation:

(1) A+R+γ = 1

Here, *A* = absorptance, *R* = reflectance, γ = transmittance.

### 2.4. Thermal Measurements

**Figure 2:**
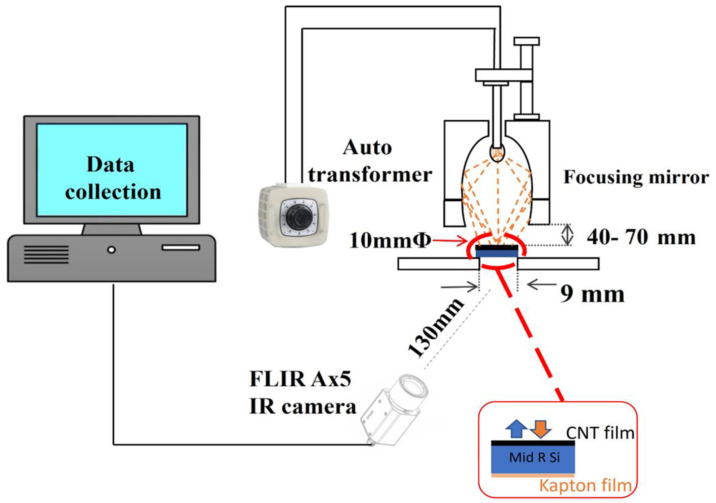
Experimental setup for lamp heating experiment involving MWCNT-coated absorbers under different intensities.

The laboratory-scale experiment employed a halogen lamp with a 10 mm beam diameter focusing mirror as an alternative source of simulated solar heat. Each MWCNT-coated sample was positioned below the heater at varying distances between ~40 and ~70 mm. A Kapton tape was attached to the rear side of each sample to monitor the output temperature using an infrared camera (FLIR Ax5 camera, Teledyne Technologies Incorporated, Thousand Oaks, CA, USA), as shown in Figure 2. Power levels of 26 W, 48 W, and 78 W were applied via an autotransformer to investigate each MWCNT-coated absorber’s temperature response to different power intensities of irradiation. The lamp intensities were calculated using Equation (2), which expresses the relationship between intensity (*I*), power (*P*), and distance (*D*) as follows:

(2) $$I=\frac{P}{{4\pi D}^{2}}$$

The intensities implemented in these experiments were 0.04, 0.08, 0.13, 0.24, and 0.39 W/cm^2^, as determined by Equation (2). In this paper, we define low intensities as those less than 0.1 W/cm^2^, equivalent to 1 sun.

## 3. Results and Discussion

### 3.1. Cell Area Analysis

**Figure 3:**
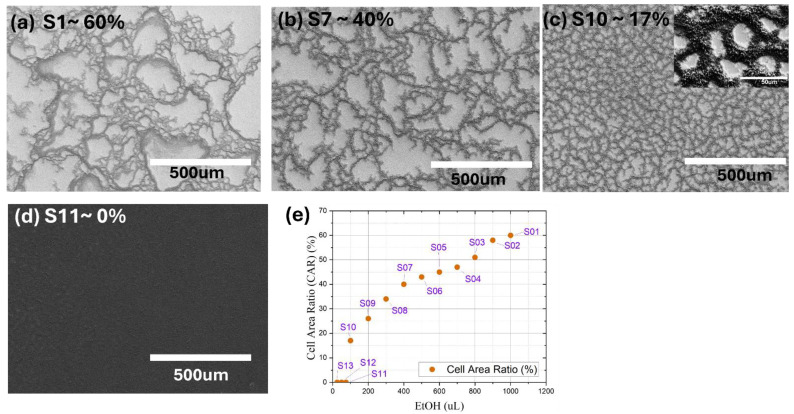
Effects of ethanol content on cell area ratio and morphology of MWCNT honeycomb structures. (**a**–**c**) FESEM images showing top surfaces of MWCNT honeycomb structures of (**a**) S1 (CAR ~ 60%), (**b**) S7 (CAR ~ 40%), (**c**) S10 (CAR ~ 17%), (**d**) S11 (CAR-0%). (**e**) Correlation between ethanol (EtOH) amount utilized in deposition process and cell area ratio (%).

The self-organized honeycomb-like structures formed by the MWCNTs are shown in Figure 3a–c. These structures are characterized by the interconnected networks of MWCNTs surrounded by pore areas. The wrinkles were formed during the thermal treatment of the MWCNT film at 300 °C to produce honeycomb structures. The formation of these wrinkles on the film results in the evaporation of the solvent [40,41].

Cell area ratio (CAR) is an essential parameter that quantifies the ratio of pore area to the total surface area of the self-organized honeycomb-structured MWCNT film. The S1, S7, S10, and S11 samples were selected for comprehensive morphological analysis, as shown in Figure 3a–d, to illustrate the range of CAR values, emphasizing the significant structural differences within the sample set. S1, having the highest CAR, demonstrates a more open structure, whereas S7 and S10 display a moderate CAR value and the lowest CAR value, respectively, and S11, with 0% CAR, signifies a completely compact network. The selected samples highlight the effect of reduced ethanol concentration on CAR and its consequent impact on the optical and thermal characteristics of MWCNT films. The graph in Figure 3e clearly represents the correlation between the amount of ethanol and the cell area ratio for each sample. As the ethanol concentration increases from 100 μL to 1000 μL, the cell area ratio increases from around ~17% to 60%. The observed pattern indicates that ethanol substantially influences the density, alignment, and porousness of the self-arranged honeycomb-structured multi-walled carbon nanotube (MWCNT) film. Higher concentrations of ethanol function as a dispersant, preventing agglomeration and facilitating the self-assembly of the nanotubes into self-organized honeycomb structures through capillary forces. As the ethanol evaporates, capillary forces induce the collapse of MWCNTs into organized patterns [42]. Higher ethanol volumes result in larger honeycomb cell areas (S01, S07) as a consequence of a more diluted dispersion, whereas lower volumes produce denser, more compact structures (S10). Additionally, reducing ethanol concentrations to below a critical threshold (100 μL) entirely prevents the formation of the honeycomb structure (S11), emphasizing ethanol’s dual role as both a dispersant and a facilitator of the forces necessary for forming the honeycomb pattern.

**Figure 4:**
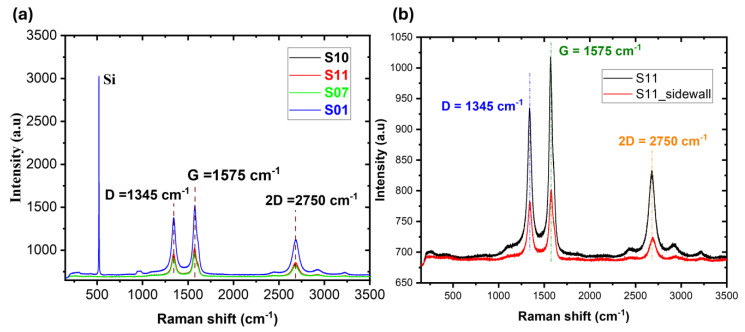
Raman spectra of MWCNT-coated absorbers on (**a**) top surfaces of S01, S07, S10, and S11, and (**b**) top surface and sidewall of fully coated MWCNT absorber of S11.

Raman spectroscopy was employed to further characterize the deposited MWCNT-coated samples. The Raman spectra of the top surfaces of the S01, S07, S10, and S11 samples are depicted in Figure 4a. Furthermore, Figure 4b illustrates the Raman spectra of the S11 sample’s top surface and sidewall. A typical silicon peak was observed at 520 cm^−1^ for the S01 sample, suggesting that the MWCNT film is not densely packed on the substrate. The G band was consistently observed at 1575 cm^−1^ and the 2D band at 2750 cm^−1^ for all samples, including the sidewall of the S11 sample, as shown in Figure 4b, while the D band of the MWCNTs was consistently observed at 1345 cm^−1^, as shown in Figure 4b. Radial breathing modes (RBM) were absent in the spectra. These spectroscopic results further demonstrate that the S11 sample exhibits complete coverage of the MWCNT film, as evidenced by the presence of a peak in the sidewall spectrum in Figure 4b, and confirm the presence of MWCNTs on the substrate.

### 3.2. Optical Analysis

**Figure 5:**
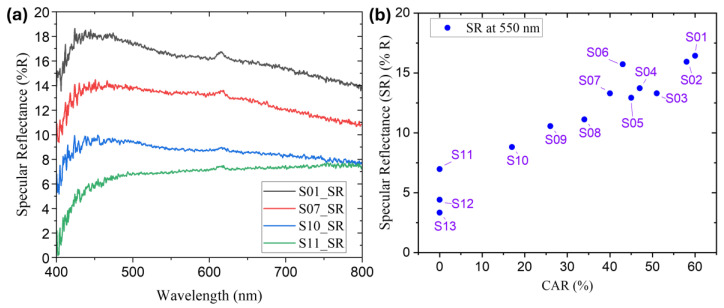
(**a**) Specular reflectance spectra at 400–800 nm of MWCNT-coated absorbers with varying cell area ratios (CARs), S01, S07, S10, and S11. (**b**) Specular reflectance at wavelength of 550 nm for MWCNT-coated absorbers.

Specular reflectance was measured in the visible spectrum from 400 nm to 800 nm using a visible spectrometer (USB2000+, Ocean Optics, USA) with a 45-degree incidence angle. Figure 5a illustrates the reflection patterns of MWCNT-coated samples with different cell area ratios (CAR), specifically S01, S07, S10, and S11. The substrate S11, which was entirely covered with MWCNT and had no CAR, demonstrated the lowest reflectance throughout the observed wavelength range.

Figure 5b provides each sample’s reflectance values at 550 nm, which were used to investigate their reflection in the peak solar intensity wavelength range (490–580 nm). The absorbers fully covered with MWCNT, designated as S11, S12, and S13, exhibited lower reflection at a wavelength of 550 nm, with reflectance values of 6.6 ± 0.7%, 4.5 ± 0.3%, and 3.3 ± 0.5%, respectively. The honeycomb-formed MWCNT absorber with the lower CAR, S10, achieved a reflection rate of 8.4 ± 0.8% at this specific wavelength. The honeycomb-structured MWCNT absorber with a higher CAR, designated as S01, demonstrated a reflection efficiency of 16.5 ± 0.8% at a wavelength of 550 nm. These findings indicate that thoroughly coating the MWCNT film resulted in the lowest reflection level across the spectrum. However, the absorber with a lower CAR still achieved a relatively low reflection level in a vital region of the solar spectrum.

**Figure 6:**
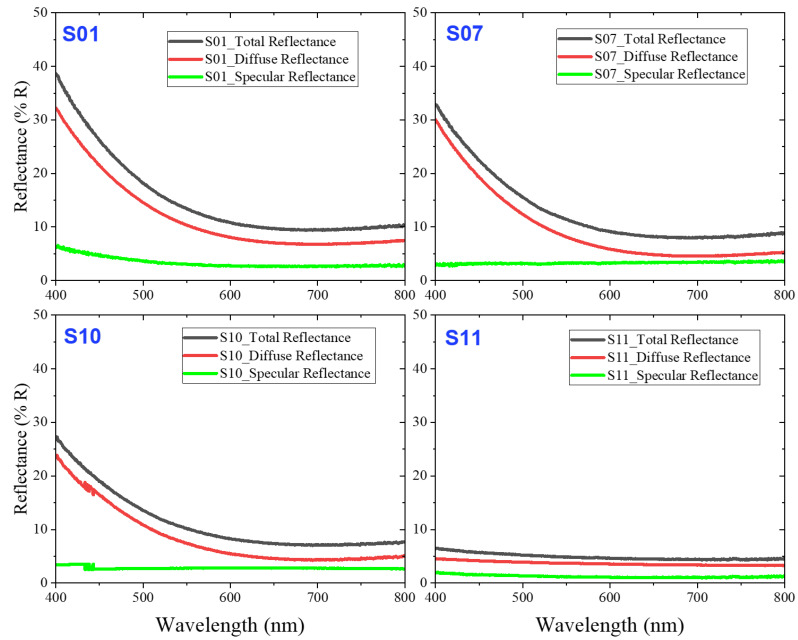
Total, diffuse, and specular reflectance of S01, S07, S10, and S11 samples in visible region.

A spectrometer equipped with an integrating sphere was utilized to measure the total, diffuse, and specular reflectance of the MWCNT-coated absorbers across the visible wavelength range (400–800 nm). Figure 6 illustrates the reflectance values for total, diffuse, and specular reflections of samples S01, S07, S10, and S11. The fully coated MWCNT absorber (S11) exhibited the lowest average total reflectance of 6.3 ± 0.3% within the visible range compared to the honeycomb-structured MWCNT absorbers. The honeycomb-structured MWCNT absorber with the lowest CAR value, S10 (~17%), demonstrated a reduced total reflectance of 27.3 ± 1.5% compared to the honeycomb-structured MWCNT-coated absorbers with higher CAR values, S01 and S07, which showed average total reflectance values of 39.5 ± 5.5% and 33.4 ± 7.5%, respectively.

**Figure 7:**
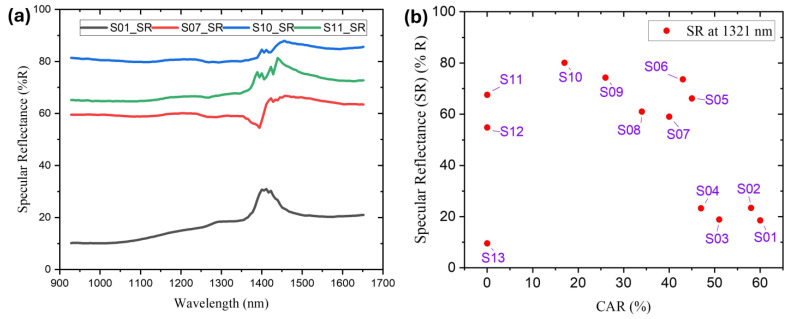
(**a**) NIR reflectance spectra of MWCNT-coated absorbers. (**b**) Effect of honeycomb-structured MWCNT-coated absorbers’ CARs on reflectance at selected near-infrared wavelength efficiency.

Furthermore, the reflectance patterns of the MWCNT-coated absorber samples in the 934–1651 nm near-infrared wavelength range were measured, as shown in Figure 7a. An IR spectrometer was employed to measure the reflection spectra in this range, and Equation (1) was used to calculate the absorbance values. All samples exhibited a minor fluctuation in reflectance between 1400 and 1450 nm, attributed to the influence of the th-SiO substrate. The absorber with the most extensive honeycomb cell area ratio (CAR) structure (S01) demonstrated the lowest reflectance throughout the entire wavelength range. Reflectance generally increased as CAR decreased (S01, S07, S10). In contrast to the sample with a honeycomb structure that exhibited a ~17% CAR (S10), the fully covered MWCNT absorber (S11) showed lower reflectance.

The reflectance values at 1321 nm are further reported in Figure 7b and were used to investigate the reflection behavior at a selected wavelength. The absorber with complete cover (S13, 0% CAR) exhibited a reflection efficiency of 11 ± 5.2% for the incident light at this specific wavelength. On the other hand, the honeycomb-structured sample identified as S10 showed the lowest CAR, at 17%, and reflected 80.5 ± 0.9% of light at a wavelength of 1321 nm. The absorber that featured a honeycomb structure and the highest CAR (60%, S01) achieved a reflection rate of 18.8 ± 3.6% at this specific wavelength. These findings indicate that absorbers with lower CARs demonstrate higher reflection in the infrared spectral range. The samples with honeycomb structures, particularly those with lower CARs, exhibit high reflectance in the infrared spectral region, highlighting potential advantages for solar thermal applications seeking to maximize thermal conversion.

**Figure 8:**
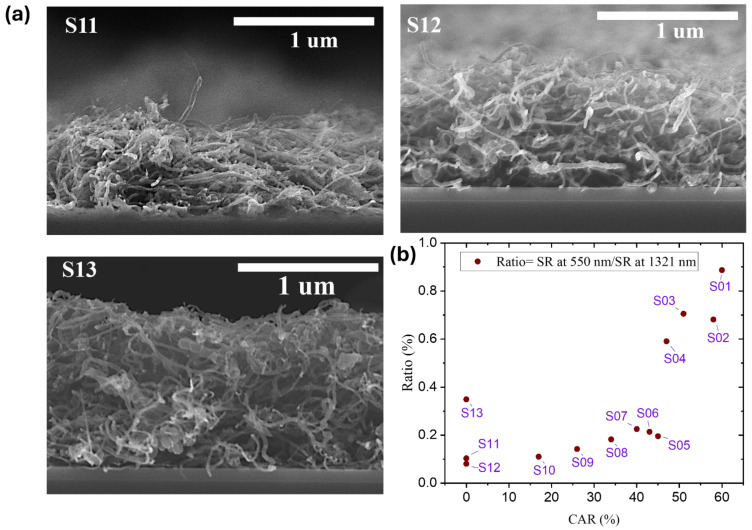
(**a**) Cross-sectional SEM images of MWCNT-coated absorbers (S11, S12, S13). (**b**) Reflectance ratios of MWCNT-coated absorbers.

The measurement of the reflection ratio at specific wavelengths, particularly the reflectance at 550 nm and 1321 nm, was performed and is graphically documented in Figure 8b. The honeycomb-structured MWCNT absorber S10 exhibited the lowest CAR value among the samples and a comparatively lower reflection ratio of 0.11%. The CAR value and the reflection ratio exhibited a distinct linear relationship, with the sample with the highest CAR (S01) exhibiting the highest reflection ratio of 0.88%. Conversely, an absorber that was completely covered in MWCNTs (S12) had the lowest reflectance ratio, at 0.08%, distinguishing it from the other absorbers fully covered with MWCNT coatings. Prior research has also shown that the density and alignment of MWCNT film influences its reflectance characteristics [43,44]. To further investigate the factors contributing to low reflectance, cross-sectional images of samples S11, S12, and S13 have been obtained and are depicted in Figure 8a. The cross-sectional images of samples S11 and S12 demonstrate a horizontal alignment of MWCNTs, identified by a non-uniform deposition and a rough surface. These findings indicate that coating uniformity is compromised at lower concentrations of MWCNTs. In contrast, the film in sample S13 shows enhanced uniformity as the MWCNT concentration increases, resulting in a more densely packed and thicker structure.

### 3.3. Thermal Analysis

MWCNT-coated absorbers efficiently absorb heat, resulting in a rise in their temperature. However, MWCNT-coated th-SiO substrates emit heat, resulting in thermal losses. The radiative heat loss adversely affects the absorber’s overall performance. The thermal conductivity of bulk MWCNT-coated film is much higher than that of th-SiO substrate due to its minimal thermal resistance and highly similar temperatures on both the top and bottom surfaces. The increased conductivity is due to the horizontal alignment and anisotropic characteristics of the MWCNT film [45]. As a result, MWCNT coating is the main factor influencing heat absorption and radiation. Additionally, the radiation loss from the MWCNT absorber is the primary focus of this investigation.

**Figure 9:**
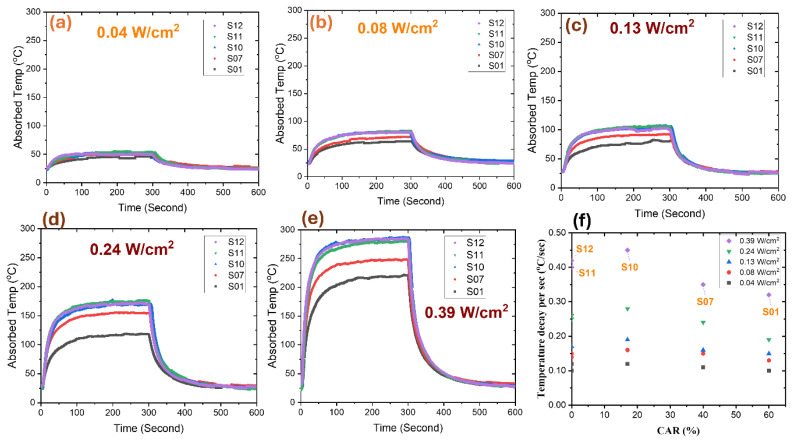
Temperature absorption profiles of MWCNT-coated absorbers (S01, S07, S10, S11, S12) under low (**a**,**b**) and high (**c**–**e**) lamp intensities. (**f**) Rates of change in temperature during lamp-off periods under different illumination intensities.

The MWCNT-coated solar absorbers were allowed to attain their maximum absorption temperature under saturated heating conditions through the operation of a halogen lamp for 300 ± 10 s. The lamp was turned off after the 300 ± 10 s heating period to allow each absorber to cool passively for 300 ± 10 s as it reverted to its initial resting position and temperature, as shown in Figure 9a–e. The temperature of every MWCNT-coated absorber increased linearly as the illumination intensity increased. The honeycomb-structured MWCNT absorber with a 17% CAR value (S10) gradually surpassed the performance of the fully covered MWCNT-coated absorber (S12) at higher intensities, despite the fact that the latter exhibited superior temperature absorption under low-intensity conditions. The observed trend has been attributed to the decreased radiative heat loss of the honeycomb structure.

The temperature decay rate for all MWCNT-coated absorbers was determined and is illustrated in Figure 9f during the lamp-off period. The temperature decay rates of the entirely concealed MWCNT absorber (S12) were measured at 0.1 ± 0.02, 0.14 ± 0.05, 0.17 ± 0.07, 0.25 ± 0.14, and 0.4 ± 0.24 °C/s, respectively, when exposed to irradiances of 0.04, 0.08, 0.13, 0.24, and 0.39 W/cm^2^. Conversely, the MWCNT absorber with a honeycomb structure and a CAR value of ~17% exhibited the most significant temperature decay rates of 0.12 ± 0.02, 0.16 ± 0.04, 0.19 ± 0.08, 0.28 ± 0.14, and 0.45 ± 0.26 °C/s when exposed to the same irradiances. Significantly, there was a positive correlation between an increase in CAR value and a decrease in temperature decay rate.

**Figure 10:**
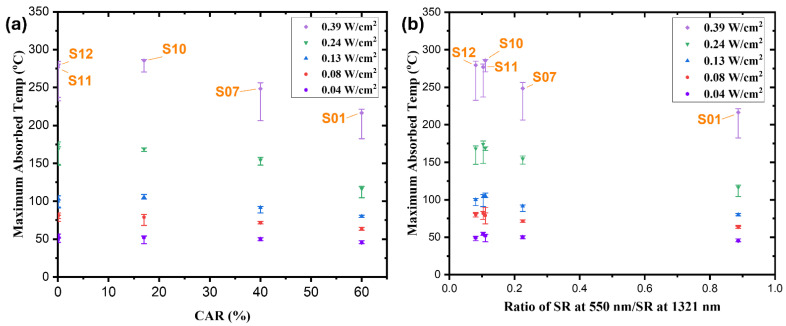
(**a**) Maximum absorbed temperatures under different lamp intensities with various CARs of S01, S07, S10, S11, and S12, and (**b**) maximum absorbed temperatures and reflectance ratios under different illumination intensities.

The relationship between the maximum absorption temperature and the honeycomb-structured absorbers’ CARs is shown in Figure 10a. In comparison to the fully covered MWCNT-coated substrates (S11, S12 with 0% CAR) and absorbers with larger honeycomb structures (S01 with 60% CAR), sample S10 absorbed the optimum temperatures of 52.5, 80.0, 105.5, 170.0, and 286.5 °C under irradiances of 0.04, 0.08, 0.13, 0.24, and 0.39 W/cm^2^, respectively. Compared to the full coatings and higher CAR values of the other MWCNT absorbers, the 17% CAR of the self-organized honeycomb-structured MWCNT-coated absorber S10 decreased radiation losses and increased absorption temperatures.

The solar thermal conversion efficacy of the self-organized honeycomb-structured MWCNT absorbers is confirmed by the relationship between the maximum absorption temperature and the reflection ratio under varying illumination intensities, as illustrated in Figure 10b. Under irradiances of 0.04, 0.08, 0.13, 0.24, and 0.39 W/cm^2^, sample S10 absorbed temperatures of 52.5, 80.0, 105.5, 170.0, and 285.5 °C, respectively, with a reflection ratio of 0.11%. The S10 sample was compared to the entirely coated MWCNT substrates S11 (reflection ratio 0.10%) and S12 (reflection ratio 0.08%), as well as the absorber S01, which had a larger honeycomb structure and a reflection ratio of 0.88%. The results indicate that the self-organized honeycomb-structured MWCNT-coated absorber with the lowest reflection ratio of 0.11% and a lower CAR value achieved the highest absorption temperatures under varying illumination intensities, confirming its superior solar thermal conversion performance. These results suggest that it can be used in low-intensity instances, such as producing hot water for baths and showers and sterilizing medical equipment. In addition, the S10 sample’s exceptional absorption is advantageous for producing electricity for low-power devices, such as sensors and small electronic equipment, and for generating potable water in coastal regions, particularly under high-intensity conditions. These applications closely align with SDG 6 [46] and SDG 7 [47].

## 4. Conclusions

In summary, MWCNT-coated absorbers were prepared to utilize the drop-casting technique, resulting in a honeycomb structure with varying cell area ratios. The optical properties of the fully coated MWCNT absorbers and the obtained structures were examined. The concentration of the MWCNT solution used in the deposition process was regulated using an EtOH solution to form self-organized honeycomb structures on thermal CVD SiO/Si substrates with well-controlled cell area ratios ranging from ~60% to 17%.

Reflectance spectra were analyzed in both visible (400–800 nm) and near-infrared (934–1651 nm) wavelength ranges. MWCNT absorbers that were utterly covered demonstrated the lowest reflection throughout the visible spectrum. However, MWCNT coatings with a honeycomb structure and a CAR value of ~17% exhibited a significant equilibrium: they maintained a reflection rate of 8.4 ± 0.8% in the visible range, specifically at a wavelength of 550 nm—which is crucial for solar energy harvesting—and exhibited 80.5 ± 0.9% reflectance at a 1321 nm wavelength. The reflection ratio between 550 nm and 1321 nm showed a linear relationship with CAR, with the lowest CAR value displaying the lowest ratio. This feature potentially increased thermal conversion efficiency by reducing infrared losses.

Furthermore, the solar thermal performance of MWCNT-coated absorbers under varying irradiance intensities, ranging from low (0.04–0.08 W/cm^2^) to high (0.13–0.39 W/cm^2^), was evaluated. The investigation showed that the honeycomb-structured MWCNT coating with a ~17% CAR performed exceptionally well at all measured intensities, reaching the maximum absorption temperatures (ranging from 52.5 to 285.5 °C) as the irradiance increased. The MWCNT-coated absorbers with ~17% CAR exhibited the highest temperature absorption compared to the fully covered MWCNT absorber and the honeycomb-structured MWCNT-coated absorbers with higher CAR values. In addition, the study found a direct relationship between the reflection ratio and the efficiency of temperature absorption at different levels of illumination intensity.

This research highlights the substantial potential for the large-scale production of cost-effective solar thermal absorbers through the application of optimized honeycomb-structured MWCNT-coated absorbers. The prospective applications of these absorbers, particularly those with a ~17% CAR value, are made possible by their superior performance in various solar thermal technologies.

## Data Availability

All data generated or analyzed during this study are included in this published article.
